# Temperature and storage time strongly affect the germination success of perennial *Euphorbia* species in Mediterranean regions

**DOI:** 10.1002/ece3.5535

**Published:** 2019-09-21

**Authors:** Antonia Cristaudo, Stefania Catara, Antonio Mingo, Alessia Restuccia, Andrea Onofri

**Affiliations:** ^1^ Department of Biological, Geological and Environmental Sciences University of Catania Catania Italy; ^2^ Department of Agricultural Sciences University of Naples Federico II Portici Italy; ^3^ Department of Agriculture, Food and Environment University of Catania Catania Italy; ^4^ Department of Agricultural, Food and Environmental Sciences University of Perugia Perugia Italy

**Keywords:** cardinal temperatures, germination strategy, perennial *Euphorbia* species, seed dormancy, storage time, thermal time, thermo‐inhibition

## Abstract

This study aims to explore the effect of environmental factors (temperature, light, storage time) on germination response and dormancy patterns in eight Mediterranean native wildplants, belonging to the *Euphorbia* L. genus. In detail, we considered *E. amygdaloides* subsp. *arbuscula*, *E. bivonae* subsp. *bivonae*, *E. ceratocarpa*, *E. characias*, *E. dendroides*, *E. melapetala*, *E. myrsinites*, and* E. rigida*. We collected seeds from natural plant populations and performed germination assays in climatic chambers at seven constant temperatures (from 5 to 35°C, with 5°C increments), and four fluctuating temperature regimes (8/15, 8/20, 8/25, and 8/30°C, with a 12/12 hr thermoperiod). Germination assays were set up both in dark (D) and in light/dark conditions (L/D, 12/12 hr photoperiod), after short and long seed storage (SS around 30 days and LS around 150 days). For all these species, except *E. amygdaloides* subsp. *arbuscula*, results show that the final germinated proportions were improved by a long storage period (>150 days), which supports the existence of nondeep physiological dormancy. Optimal temperature levels ranged from 14.3 to 21.3°C and base temperatures ranged from 5.6 to 12.1°C, while ceiling temperatures from 25.6 to 34.7°C. For none of these species, germinations were favored by an alternating daily temperature regime, while in several instances, germinations were quicker and more complete in darkness, than in an alternating light/dark regime. In some instances, extreme temperature levels (5 and 30°C) induced dormancy and germinations did not resume when seeds were exposed at optimal temperature levels. Results are discussed in terms of the dynamics of emergences and how this might be affected by climate changes.

## Graphical abstract

Most of the *Euphorbia* species under investigation showed physiological dormancy, which was released following an after‐ripening period (nondeep Physiological Dormancy; PD). On average, germinations were quickest and most complete at temperature levels ranging from 14 to 21°C, while there was no or little effect of light and alternating temperature regimes. The germination of most Euphorbia species was obstacled at temperatures as low as 5°C and as high as 35°C, due to either thermo‐inhibition or thermo‐dormancy. Under present climatic conditions, germination occurs mainly in the autumn season. Due to thermal requirements, the germination of these species may be vulnerable to climate change, which may narrow the favorable germination season and affect the dynamics of plant populations.

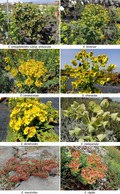

## 1 INTRODUCTION

Seed germination behavior is a fundamental trait to determine the ability of plants to successfully establish in a given habitat or geographical region. Indeed, seeds need to be able to germinate in the right timing and location, so that the chances of seedling survival and establishment are maximized. A key role is played by several environmental factors, including temperature (*T*), light, and water availability. In particular, the effect of temperature is related to threshold levels for seed germination, such as base temperature (*T*_b_), optimum temperature (*T*_o_), and maximum or ceiling temperature (*T*_c_), which form the basis for the concept of thermal time.

Germination is inhibited for *T* < *T*_b_ and *T* > *T*_c_ (thermo‐inhibition). Thermo‐inhibition at high temperatures is very important for many perennial species or winter annual species adapted to warm climates, which have an optimum germination temperature between 10 and 20°C (Thanos, Georghiou, & Skarou, 1989; Thompson, 1973; Washitani & Masuda, 1990). Thermo‐inhibition is also very common in species of Mediterranean ecosystems, as well as in *Euphorbia* species inhabiting warm seasonal ecosystems (Lavorel, Debussche, Lebreton, & Lepart, 1993; Washitani & Masuda, 1990). In some cases, high (and low) temperatures can also induce secondary dormancy, according to a phenomenon known as thermo‐dormancy (Bewley & Black, 1985). This was observed especially in annual plants of deserts as well as in some Mediterranean species. In both cases, thermo‐dormancy could play a strategic role (Maher, Gerasopoulos, & Maloupa, 2000; Thanos et al., 1989) in preventing seed germination during occasional rain in hot and dry summers (Gutterman, 2002). Thermo‐dormancy has also been found in seeds of *Lactuca sativa* and *Euphorbia nicaeensis* (Narbona, Arista, & Ortiz, 2007a; Thanos et al., 1989; Vidaver & Hsiao, 1975).

Apart from the prevailing temperature level, thermal history also plays an important role, in the sense that several plant species may prefer a fluctuating temperature regime and may not germinate with constant daily temperatures (Cristaudo, Gresta, Catara, & Mingo, 2014; Masin, Onofri, Gasparini, & Zanin, 2017).

Light is also important, and, in this respect, previous research has shown that some plants give better germination capabilities when exposed to an alternating light/dark regime (Catara et al., 2016).

In addition to the climatic variables, germination is also controlled by endogenous factors, such as primary dormancy. Although this is a potentially expensive trait (Willis et al., 2014), it has been recognized as an adaptive mechanism, by which the plant avoids germination in unfavorable conditions for seedling establishment (e.g., excess heat and drought) and postpones them until the season is more favorable (Baskin & Baskin, 2014).

Among the endogenous factors, several studies have shown that seed germination is genetically determined and the phylogenetic signal may be a significant constraint to interspecific variation within a genus (Zhang, Du, & Chen, 2004). This was found, for example, for some *Romulea* species in Mediterranean habitats (Carta, Hanson, & Müller, 2016), even though a contrasting behavior was observed with other genera, such as *Stellaria* and *Nothofagus*, which showed considerable interspecific variability in germination behavior (Arana et al., 2016; Vandelook, Van de Moer, & Van Assche, 2008).

Morphological traits, such as seed size, seed mass, seed shape, and seed dispersal, can also play a role in seed germination behavior as shown, for example, in *Verbascum* sp. pl., *Euphorbia humifusa* and *Solanum nigrum* (Catara et al., 2016; Wang et al., 2016).

Studying the relationship between environmental factors and plant physiology is fundamental to understand and predict ecological dynamics, depending on the climate characteristics of a certain location, such as latitude, altitude, soil moisture, temperature and rainfall patterns, light, and photoperiod (Baskin & Baskin, 2014; Cristaudo, Gresta, Restuccia, Catara, & Onofri, 2016; Gresta, Cristaudo, Onofri, Restuccia, & Avola, 2010; Zhang et al., 2017). This is particularly important for Mediterranean regions, which are characterized by a high variability of environmental conditions, where wet and mild periods are alternated with dry and hot periods, which make seedling survival very difficult. In this respect, climate change and global warming should be carefully considered, as they can pose additional problems for seed germination and plant recruitment (Baskin & Baskin, 2014; Mondoni, Rossi, Orsenigo, & Probert, 2012; Walck, Hidayati, Dixon, Thompson, & Poschlod, 2011), which can modify the geographical distribution of plant species (Poschlod et al., 2013).

Climate change has been shown to produce a more erratic rainfall pattern, with higher temperatures and more frequent conditions of water shortage. A few studies have addressed the effects of water stress on germination in Mediterranean species. Conifers have shown high tolerance to water stress (Boydak, Dirik, Tilki, & Çalikoğlu, 2003; Thanos & Skordilis, 1987), whereas shrub species have shown variable responses, from high (e.g., *Antyllis cytisoides*; Ibanez & Passera, 1997) to low or moderate tolerance (e.g., *Genista scorpius*, *Cistus monspeliensis*, *C. salviifolius*, *Calicotome villosa*; Bochet, García‐Fayos, Alborch, & Tormo, 2007; Chamorro, Luna, & Moreno, 2017; Pérez‐Fernández, Calvo‐Magro, & Ferrer‐Castán, 2006). Negative effects of water stress can persist for several years, through maternal effects which lead to less viable seeds with lowest germination capacity. Unfortunately, the effects of climate change on seed germination seem to be highly variable, depending on plant species, populations, and sites, which makes it difficult to make predictions for a given species at a specific site. This motivates research on the germination responses to climate factors, such as temperature, light, and water availability.

In this study, we present a novel assessment of seed germination strategies, focusing on some taxa of the *Euphorbia* L. genus (Euphorbiaceae Juss.). This is the largest genera of the Euphorbiaceae family, also known as spurge family, and it is one of the five most species‐rich genera of the angiosperm group, with around 2,000 species (Frodin, 2004; Govaerts, Frodin, & Radcliffe‐Smith, 2000), that occur in all temperate and tropical regions. Some of them have a considerable economic importance in medicine (Rahman & Akter, 2013) or for revegetation, landscaping, and xero‐gardening in semiarid environments (Benvenuti, 2014; Franco, Martínez‐Sánchez, Fernández, & Bañón, 2005).

**Figure 1:**
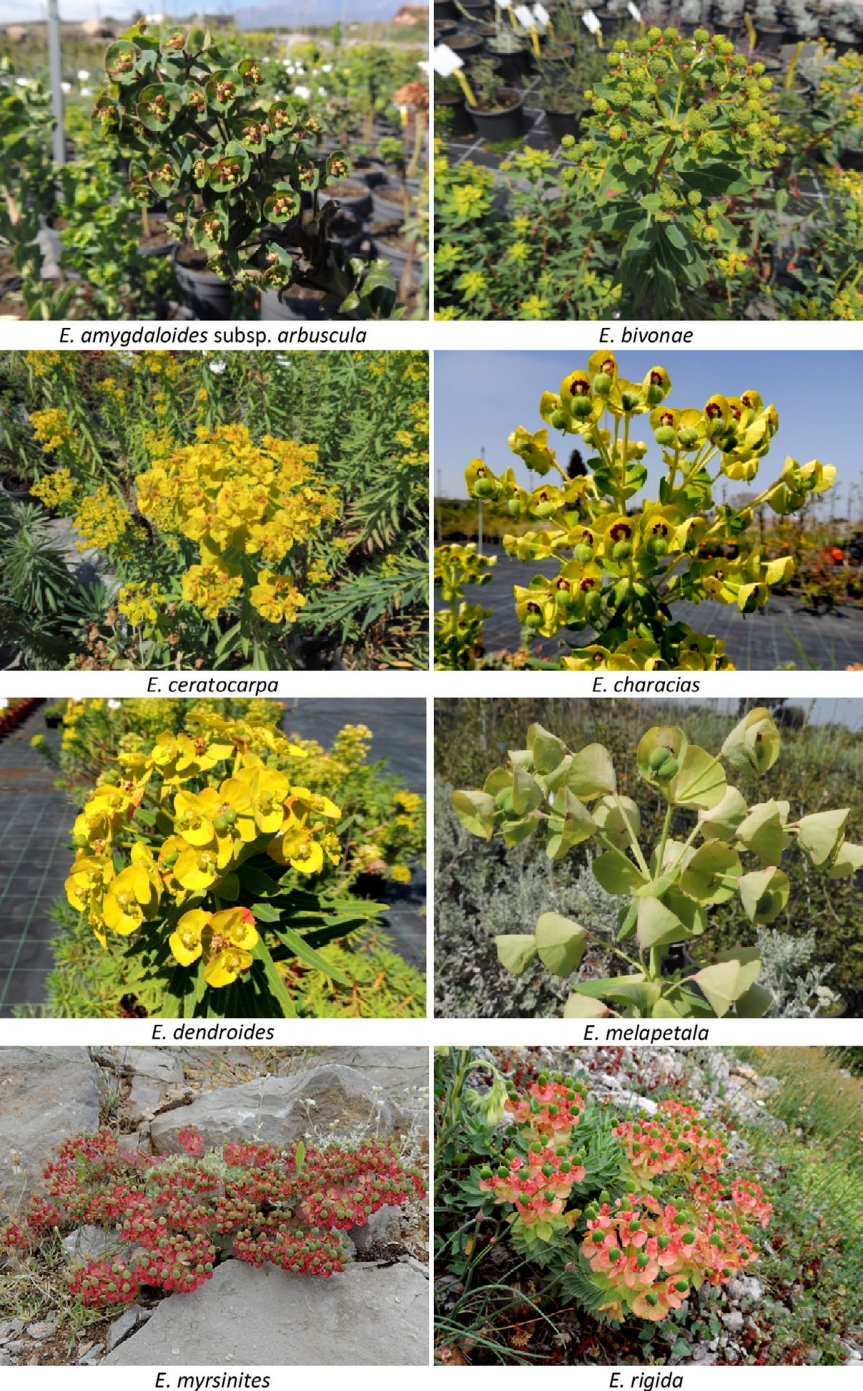
Photographs of the eight *Euphorbia* study species

We focused on eight perennial taxa, on which no pertinent information about the germination traits is available from literature: *Euphorbia amygdaloides* L. subsp. *arbuscula* Meusel, *E. bivonae* Steud. subsp. *bivonae*, *E. ceratocarpa* Ten., *E. characias* L., *E. dendroides* L., *E. melapetala* Gasp. ex Guss., *E. myrsinites* L., and *E. rigida* M. Bieb. (Figure 1). The investigated species are phylogenetically related and belong to the monophyletic subgenus *Esula* Pers., one of the four major clades within the genus *Euphorbia* (Geltman, 2015; Riina et al., 2013). Some of these species are widespread across the Mediterranean region, in a wide range of altitudes, while three of them are narrowly distributed (endemic).

**Figure 2:**
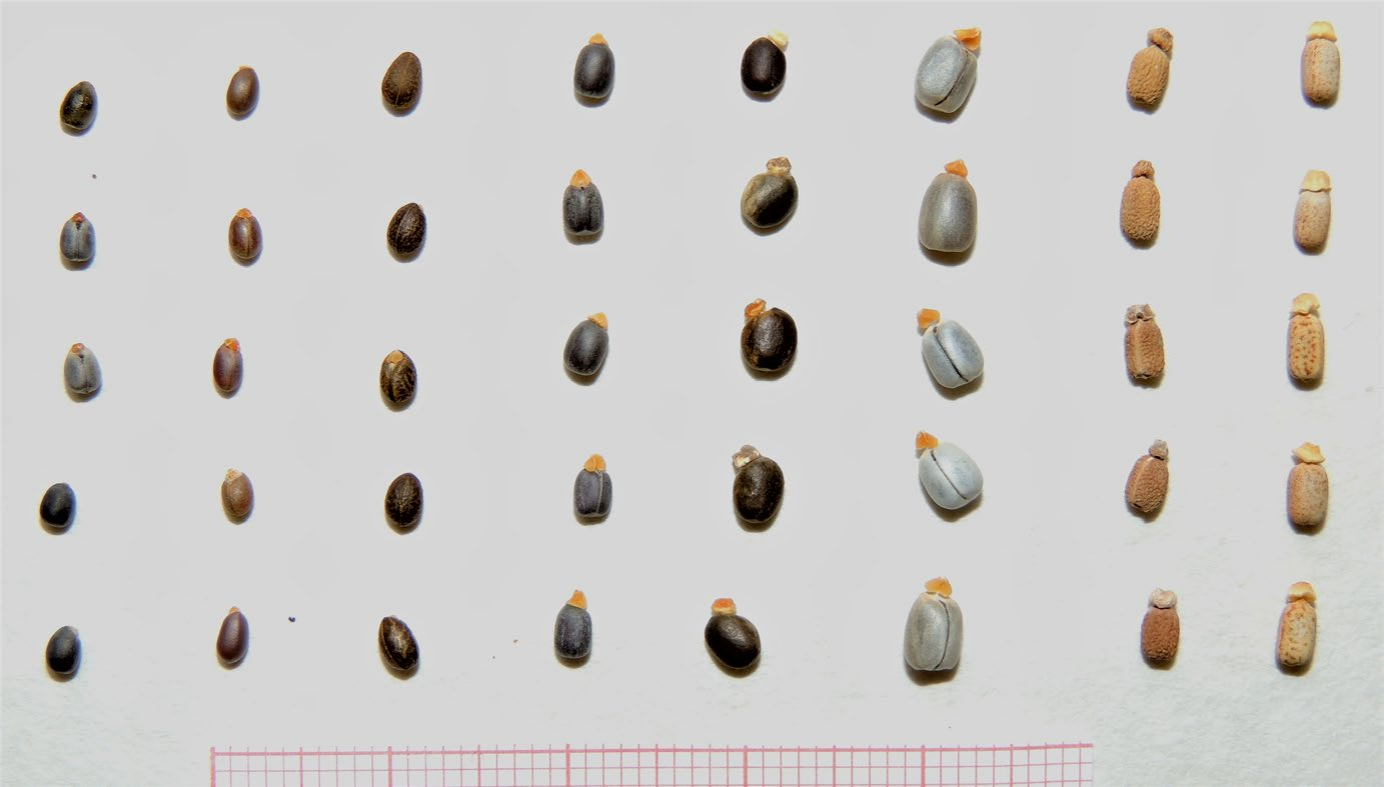
Photograph about seeds of *Euphorbia* study species

The seeds of all species in the group possess a caruncle, but differences in the size and shape of the caruncle are pronounced. Seed can have different sizes and, above all, different shapes (ovoidal, quadrangular or strongly compressed laterally), ornamentation (smooth or vermiculate‐rugulose) and color (brown or gray) (Figure 2).

Some of these species, such as *E. characias*, *E. dendroides*, *E. myrsinites*, and *E. rigida*, are characterized by interesting biotechnical and/or ornamental value, both for architectural shapes and fascinating foliage (La Mantia et al., 2012; Pahlevani, Geltman, & Riina, 2011; Riina et al., 2013; Sari & Karaşah, 2015).

Considering those eight species, the objectives of this study were as follows: (a) evaluate the relationships between temperature, light, storage time, and germination behavior; (b) estimate threshold temperatures for seed germination and thermal time; and (c) verify the existence of thermo‐inhibition and/or thermo‐dormancy.

## 2 MATERIALS AND METHODS

### 2.1 Fruit collection and seed storage

Seeds were collected in four provinces of Eastern and Western Sicily (Catania, Messina, Palermo, Trapani) where the plants are abundant under wild conditions. The main characteristics of the species under investigation are reported in Table 1.

**Table 1:** Chorological, ecological, environmental, and morphological characteristics of the *Euphorbia* species under investigation

Species	Chorological type	Life form	Habitat	Altitudinal range (m a.s.l)	Environmental characteristics			Seed mass (mg per seed) (1)
					Climate	Light	Soil moisture	
*E. amygdaloides* subsp. *arbuscula*	Endemic to Sicily, Sardinia and Calabria	Chamaephytes perennial herb, woody at the base	Oak and beech‐woods	600–1,500	Temperate (cool and moist)	Partial shade	Moist	4.38 (0.17)
*E. bivonae*	Stenomediterranean	Nanophanerophytes perennial woody	Rocky ridges of the coastal lands, calcareous rocks	0–300	Mediterranean (hot and dry)	Full sun	Dry	5.04 (0.05)
*E. ceratocarpa*	Endemic to Sicily and southern Italy	Chamaephytes perennial herb, woody at the base	Disturbed soils, road edges	0–850	Intermediate between Mediterranean and temperate	Full sun	Dry to moist	5.78 (0.13)
*E. characias*	Stenomediterranean	Nanophanerophytes perennial woody	*Quercus ilex* woods, scrublands, garrigues	0–1,000	Mediterranean	Full sun to partial shade	Dry	9.63 (0.37)
*E. dendroides*	Stenomediterranean	Nanophanerophytes perennial woody	Rocky limestone and volcanic slopes	0–800	Mediterranean	Full sun	Dry	9.61 (0.35)
*E. melapetala*	Endemic to Sicily and Malta	Nanophanerophytes perennial woody	Scrublands, dry slopes	150–1,400	From Mediterranean to temperate	Partial shade	Dry to moist	17.49 (0.57)
*E. myrsinites*	S‐European‐Asia Minor	Chamaephytes perennial herb, woody at the base	Mountain pastures, rocky dry slopes, calcareous slopes	600–1,900	From Mediterranean to temperate	Full sun	Dry	8.96 (0.12)
*E. rigida*	S‐European	Chamaephytes perennial herb, woody at the base	Dry rocky, exposed, limestone and volcanic slopes; dry river beds	400–1,500	From Mediterranean to temperate	Full sun	Dry	10.70 (0.21)

Nomenclature of plant species follows the Planetary Biodiversity Inventory (PBI) *Euphorbia* database (http://app.tolkin.org/projects/72/taxa; Riina & Berry, 2017).

**Figure 3:**
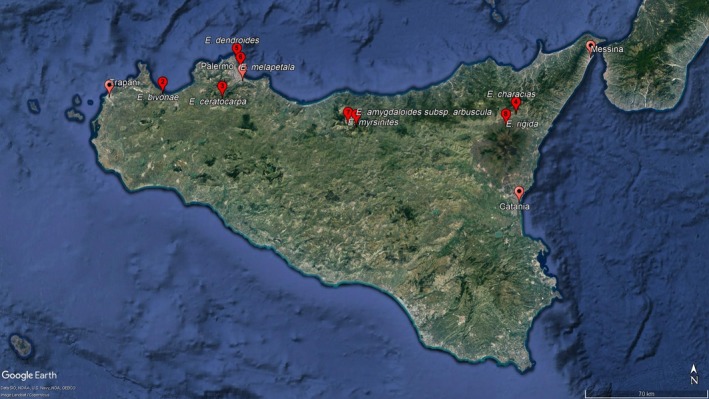
Map of sampling sites for the eight study species across northern Sicily. Numbers coincide with site numbers in Table 2

Seeds of all eight *Euphorbia* species were collected from native populations (Table 2, Figure 3), during May, June, and July 2012, 2013, and 2014, from mature capsules (schizocarp fruits, called cocci) ready for dispersal. Only one harvest was made for each species; seeds were immediately stored and submitted to germination tests. Large and vigorous populations were selected, with highest densities and proportions of reproductive output. A GPS (iFINDER HUNT™, Lowrance Electronics Inc.) was used to record positioning and altitude for each collection site (Table 2).

**Table 2:** Characteristics of the sampling sites, for each of the eight *Euphorbia* species

Species	Date of collection	Province	District	Collection sites	Altitude (m a.s.l.)	Geographical coordinates WGS84
1. *E. amygdaloides* subsp**.** *arbuscula*	16/06/2013	Palermo	Petralia Sottana	P.no di Farina (Madonie mountains)	1,381	37 51 42,73 N 14 04 43,78 E
2. *E. bivonae*	14/05/2013	Trapani	Castellammare del Golfo	Mt. Inici	230	38 01 48,00 N 12 52 22,75 E
3. *E. ceratocarpa*	25/07/2013	Palermo	Monreale	Contrada Strasatto	830	38 00 26,4 N 13 14 49,2 E
4. *E. characias*	26/05/2012	Messina	Francavilla di Sicilia	C.da Serro Piddu	570	37 54 40,22 N 15 04 21,41 E
5. *E. dendroides*	14/05/2013	Palermo	Palermo	Mt. Pellegrino	39	38 11 40,70 N 13 20 17,08 E
6. *E. melapetala*	14/05/2014	Palermo	Palermo	Mt. Pellegrino	140	38 11 09,20 N 13 20 43,35 E
7. *E. myrsinites*	16/06/2013	Palermo	Petralia Sottana	P.no Battaglia (Madonie mountains)	1,636	37 52 27,60 N 14 01 41,98 E
8. *E. rigida*	27/05/2012	Catania	Castiglione di Sicilia	Regional road “Mareneve” (Etna volcano)	956	37 51 15,62 N 15 00 31,75 E

Fruits were collected from randomly chosen individuals (>50 individuals per species) in a relatively big area (200–500 m^2^) to obtain an adequate representation of genetic diversity. Plants were randomly selected from the middle of each population, in order to avoid any edge effects and any disturbances to the natural spread of the population (sustainable harvesting).

In the laboratory, the fruits were air‐dried at room temperature (25°C ± 2°C), for about 3 weeks, in plastic box perforated for ventilation with bottom surfaces covered with blotting paper, to absorb humidity; mesh lids were used, to ensure air circulation and prevent seed losses, due to the “explosion” of fruits. After their release, seeds were cleaned from the remaining fruit tissues using a stack of sieves. For each taxon, the weight of five replicates of 20 randomly chosen seeds was determined (Table 1). The average weight of one dry seed, expressed in mg, of each species was determined using a precision balance with an accuracy of 0.0001 g (Mettler AE 50). A preliminary cut test on a subsample of seeds showed that the viability was nearly 100%, for all species. The collected seeds were stored in paper bags in laboratory conditions (22 ± 2°C, 50% RH), until the beginning of the germination experiments, starting approximately 1 month from the collection, for all species, regardless of the sampling year.

### 2.2 Experimental design

Germination experiments were carried out in automatic temperature‐, humidity‐, and light‐controlled growth chambers (Sanyo ‐ model MLR‐351H). Light was delivered via cool white fluorescent tubes (Osram FL 40 SS W/37), with a photon flux density of 50 μmol m^−2^s^−1^.

For all species, we considered seven constant temperature regimes (from 5 to 35°C, with 5°C increments) and four fluctuating temperature regimes (8/15, 8/20, 8/25, and 8/30°C) with a 12/12 hr thermoperiod. A further constant temperature treatment at 8°C was added as control for fluctuating temperatures. Temperature regimes were chosen considering the typical Mediterranean conditions, where 10°C corresponds to the average daytime temperature in late autumn or winter, while 20°C corresponds to the average daytime temperature early in autumn and late in spring. The highest temperatures (30 and 35°C) are common in Mediterranean summer conditions, and they were included to test for possible thermo‐inhibition or thermo‐dormancy phenomena in imbibed seeds. Maximum and minimum daily temperatures for fluctuating regimes were selected to represent the typical extent of daily fluctuations.

All regimes were set up in continuous darkness (D) and in light/dark conditions (L/D, 12/12 hr photoperiod, with the lowest temperature during the dark period).

Germination assays were repeated twice, considering the same populations at two different storage times: short storage (SS: around 30 days) and long storage (LS: around 150 days). Germination assays at fluctuating temperatures were performed only after long storage.

For each germination assay, four replicates of 25 randomly selected seeds were used for each treatment and studied species. Seeds were placed in 9 cm diameter Petri dishes, on top of three layers of filter papers (Whatmann No. 1), previously moistened with 5 ml of distilled water and incubated in growth chambers at the different temperature regimes.

For darkness treatments, the Petri dishes were wrapped in two layers of aluminum foil and kept in the same growth chamber. All Petri dishes were sealed using Parafilm^®^ to avoid moisture losses; water was added to the dishes as needed to keep an adequate moisture level.

Seed germination was monitored daily, and germinated seeds were counted and removed from Petri dishes. Seeds were considered as germinated when the radicle protrusion was about 2 mm. In continuous darkness treatments, counts were made under green‐filtered light (Philips PF710E), which had not shown any effect on seed germination, as assessed by preliminary assays. In L/D, germination counts were performed during the light period. Experiments were continued for 30 days. At the end of incubation period, the viability of the remaining seeds was estimated by cut test; seeds with white, hard embryos were considered to be alive. Empty and dead seeds were excluded from the calculation of final germination percentages.

After the end of assays, ungerminated seeds exposed at 5 and 30°C for six *Euphorbia* species (*E. amygdaloides* subsp. *arbuscula*, *E. bivonae* subsp. *bivonae*, *E. ceratocarpa*, *E. characias*, *E. dendroides*, and* E. rigida*) were moved to a growth chamber at 20°C (close to optimal for all species), to assess whether they could recover germination or whether they had become dormant.

### 2.3 Data analyses

For each Petri dish, the observed counts were used to parameterize a log‐logistic germination model, by using a time‐to‐event modeling platform (Onofri, Benincasa, Mesgaran, & Ritz, 2018; Ritz, Pipper, & Streibig, 2013). The fitted model was used to derive the final percentage of germinated seeds (FPG) and the time to 50% germination (*T*_50_), which were submitted to ANOVA. FPGs were arcsine‐square‐root transformed and *T*_50_ were log‐transformed prior to analyses, in order to meet the basic assumptions for linear models; back‐transformed means were used for tables and graphs.

Considering the assays at constant temperature, the number of days to achieve 10% and 30% germination was also derived from the fitted germination models and their reciprocal values, together with the reciprocal of *T*_50_ values, were taken as the germination rates respectively for the 10th percentile (GR_10_), 30th percentile (GR_30_), and 50th percentile (GR_50_) (Bierhuizen & Wagenvoort, 1974).

The observed GRs for each species, light regime, and storage time were used to parameterize the following thermal‐time model, derived from Mesgaran, Onofri, Mashhadi, and Cousens (2017):

$${\text{GR}}_{g}=\left\{\begin{array}{cc} \frac{T-T_{b}}{\Theta_{T}\left(g\right)}\left[1-k\left(T-T_{b}\right)\right] & \text{if}\;T_{b}<T<T_{c}\\ 0 & \text{if}\;T\leq T_{b}\;or\;T\geq T_{c} \end{array}\right.$$

where *g* is the percentile (10, 30, or 50th), *T* is the temperature, Θ*_T_*(*g*) is the thermal time to germination for the *g*‐th percentile, *T*_b_ is the base temperature, and *k* relates to the decrease of germination velocity when temperature exceeds the optimal level.

## 3 RESULTS

### 3.1 Germination capability

**Figure 4:**
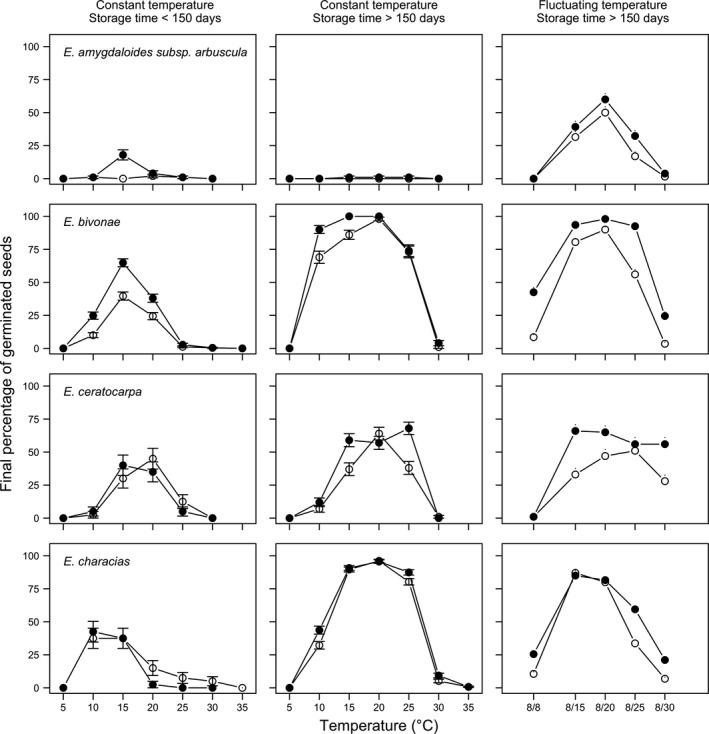
Final germination percentages of *Euphorbia amygdaloides* subsp. *arbuscula*, *E. bivonae*, *E. ceratocarpa*, and *E. characias* at two storage periods (<150 and >150 days), two light conditions (photoperiod of 12/12 hr, with the highest temperature during daytime; darkness), at constant and fluctuating temperature. (LEGEND: open symbols represent the alternating light/darkness regime while closed symbols represent the continuous darkness regime). Vertical bars represent standard errors

**Figure 5:**
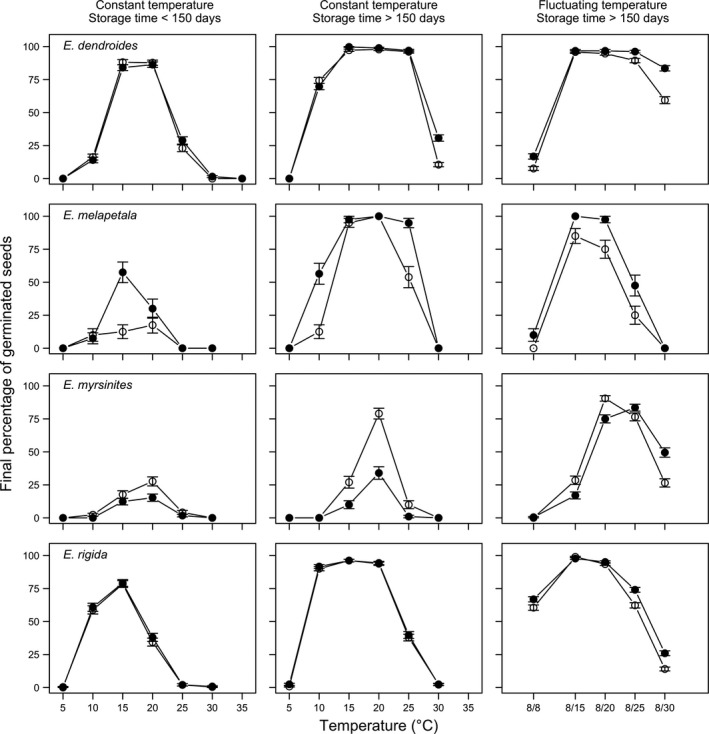
Final germination percentages of *Euphorbia dendroides*, *E. melapetala*, *E.  myrsinites*, and *E. rigida* at two storage periods (<150 and >150 days), two light conditions (photoperiod of 12/12 hr, with the highest temperature during daytime; darkness), at constant and fluctuating temperature (LEGEND: open symbols represent the alternating light/darkness regime while closed symbols represent the continuous darkness regime). Vertical bars represent standard errors

For all species, the ANOVA showed that germination capability was significantly influenced by temperature regimes, light conditions, and storage times. Germination occurred across a relatively large range of temperatures, although this appeared to be species‐dependent. Some species germinated from 15 to 25°C (*E. ceratocarpa*, *E. characias*, *E. melapetala*), and other species germinated from 10 to 25°C (*E. bivonae*, *E. dendroides*), while one species germinated between 10 and 20°C (*E. rigida*) and another one germinated only at 20°C (*E. myrsinites*; Figures 4 and 5).

In general, all species reached the highest germination capability from 15 to 20°C. Outside this range, the capability was progressively reduced, as temperature increased/decreased. It is important to note that *E. amygdaloides* subsp. *arbuscula* showed little or no germination at constant temperatures (<18%; Figure 4).

Differences between light regimes were rather small, although most species (except *E. characias* and *E. myrsinites*) gave slightly higher FGPs in continuous darkness, especially at suboptimal or supra‐optimal temperature levels (Figures 4 and 5). Differences were most visible in *E. bivonae* (especially at short storage time and with fluctuating temperature), *E. ceratocarpa*, and* E. melapetala*.

As for storage time, FGPs were often higher with long storage. Notable effects were noted with *E. bivonae*, which gave higher FGPs with long storage time, together with a wider temperature range for germination (10–25°C). Improved germination with long storage was also noted with *E. ceratocarpa*, *E. characias*, *E. melapetala*, and *E. myrsinites* (Figures 4 and 5). For this latter species, a high proportion of seeds were dormant at all constant temperatures with short storage.

Considering fluctuating temperatures, the effects were rather small, although higher FGPs were noted with *E. amygdaloides* subsp. *arbuscula* and *E. myrsinites* (Figures 3 and 4).

### 3.2 Thermo‐dormancy and/or thermo‐inhibition

**Figure 6:**
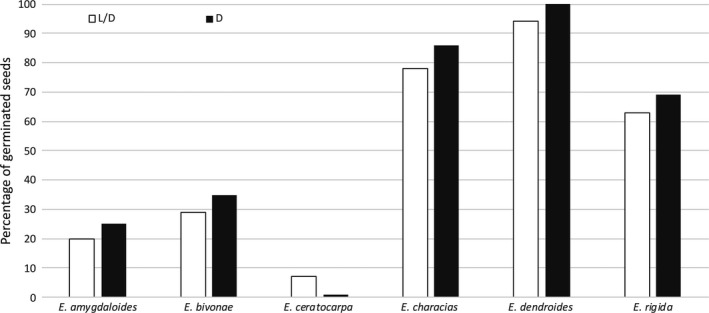
Germination of nongerminated seeds of six *Euphorbia* species after 30 days of exposure at the benefit temperature (20°C). Seeds were first exposed at 5°C for 30 days, where no germinations were observed (see Figures 3 and 4). Immediately afterward, seeds were reincubated at 20°C for 30 days

For all species, no germination was observed at 5°C for 30 days. However, when seeds were reincubated at 20°C (benefit temperature) under the same light conditions (L/D and D), *E. characias*, *E. dendroides*, and* E. rigida* exhibited a high germination capability, with FPG values ranging from 60% to 100%, both in L/D and in D. *E. amygdaloides* subsp. *arbuscula*, *E. bivonae*, and *E. ceratocarpa* showed the ability to germinate, although the FGPs were rather low (Figure 6).

**Figure 7:**
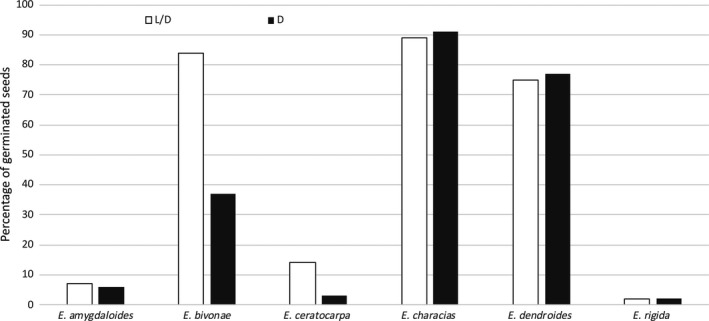
Germination of nongerminated seeds of six *Euphorbia* species after 30 days of exposure at benefit temperature (20°C). Seeds were first exposed at 30°C for 30 days, where no germinations were observed (see Figures 3 and 4). Immediately afterward, seeds were reincubated at 20°C for 30 days

Considering 30°C, for all species the germination capacity was rather low or null. When ungerminated seeds were reincubated at 20°C under the same light conditions (L/D or D), *E. bivonae*, *E. characias*, and *E. dendroides* showed high germination capacities (>80°C) while *E. amygdaloides* subsp. *arbuscula*, *E. ceratocarpa* and *E. rigida*, although viable, showed little or no germination (Figure 7).

### 3.3 Germination velocity

**Figure 8:**
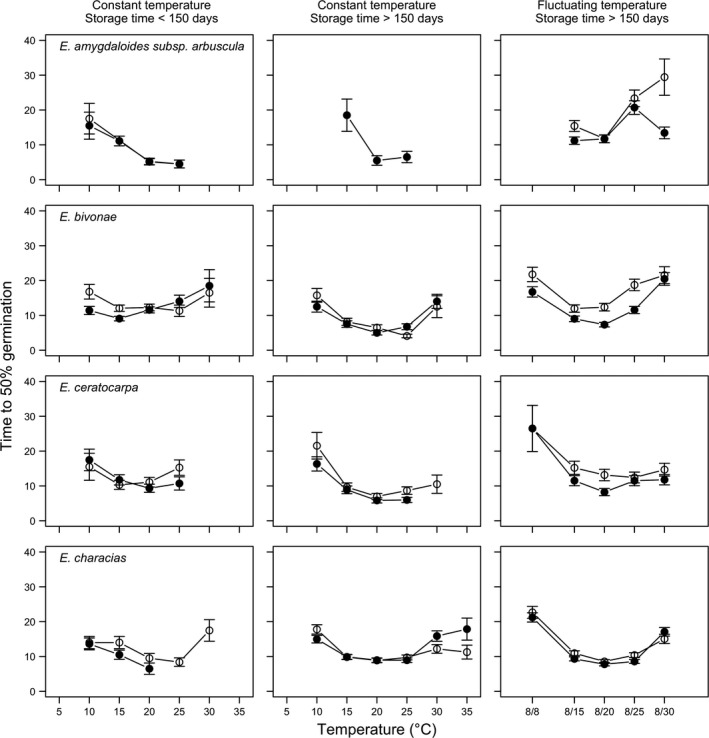
Time to 50% germination (relative to FGP) for *Euphorbia amygdaloides* subsp. *arbuscula*, *E. bivonae*, *E. ceratocarpa*, and *E. characias* at two storage periods (<150 and >150 days), two light conditions (photoperiod of 12/12 hr, with the highest temperature during daytime; darkness), at constant and fluctuating temperature. (LEGEND: open symbols represent the alternating light/darkness regime while closed symbols represent the continuous darkness regime). Vertical bars represent standard errors

**Figure 9:**
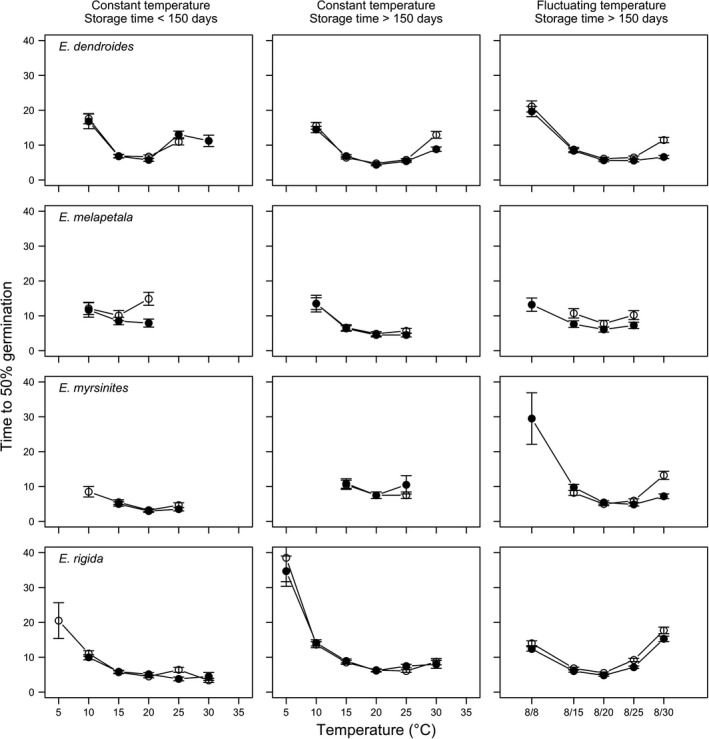
Time to 50% germination (relative to FGP) for *Euphorbia dendroides*, *E. melapetala*, *E. myrsinites*, and *E. rigida* at two storage periods (<150 and >150 days), two light conditions (photoperiod of 12/12 hr, with the highest temperature during daytime; darkness), at constant and fluctuating temperature (LEGEND: open symbols represent the alternating light/darkness regime while closed symbols represent the continuous darkness regime). Vertical bars represent standard errors

Considering the germinated fraction, the time (days) taken for 50% of seeds to germinate (*T*_50_) generally decreased as temperature increased from 5 to 20°C (Figures 8 and 9). All species showed quickest germinations between 15 and 20°C, requiring <10 days to reach 50% germination. On average, the ranking among species at constant 20°C, short storage, and in dark conditions was: *E. myrsinites* < *E. rigida* < *E. dendroides* < *E. characias* < *E. melapetala* < *E. ceratocarpa* < *E. bivonae* with, respectively, 3.0 (*SE* = 0.37), 5.1 (0.44), 5.8 (0.42), 6.5 (1.63) 7.9 (1.15), 9.3 (1.17), and 11.6 (0.87) days. For all species, *T*_50_ was remained constant or increased slightly in alternating light conditions, although such an effect was significant only with *E. characias* (from 6.5 to 9.5 days) and, particularly with *E. melapetala* (from 7.9 to 14.9 days). A long storage time caused an increase of *T*_50_ in dark conditions for *E. myrsinitis* (from 3.0 to 7.5 days) and a decrease for *E. melapetala* (from 7.9 to 4.5 days), *E. ceratocarpa* (from 9.3 to 5.8 days), and *E. bivonae* (from 11.6 to 5.0 days).

### 3.4 Thermal‐time parameters

Threshold temperatures (*T*_b_, *T*_o,_ and *T*_c_) are reported in Table 3. In general, in almost all the *Euphorbia* species with long storage, base, optimal, and ceiling temperatures were lower than with short storage, both in continuous darkness and alternating light regime. *T*_b_ values showed some variability among tested species, ranging from −0.5°C (*E. characias*) to 13°C (*E. myrsinites*). The *T*_b_ for germination of *E. bivonae*, *E. dendroides*, and *E. ceratocarpa* indicated that these three species were able to germinate already at temperatures ranging from 7.6 to 9.5°C. *E. myrsinites* required warmer temperatures (13°C), and *E. rigida*, which is usually found in wide altitudinal range, started germinating at cooler temperatures (6.2°C). *T*_o_ values fall in a relatively narrow interval, ranging approximately from 14.3 to 21.3°C, according to the species, light conditions, and storage time. *T*_c_ changed from a minimum of 21.1°C (*E. characias* in the L/D regime) to a maximum of 34.3 (*E. characias* in the L/D regime), in short storage, and from a minimum of 25.6°C (*E. myrsinites*) to a maximum of 34.7°C (*E. dendroides*), in long storage.

**Table 3:** Cardinal temperatures for germination for seven *Euphorbia* species exposed to a constant temperature regime (*T*_b_: base temperature; *T*_c_: ceiling temperature; *T*_o_: optimal temperature), together with thermal times to germination for the 10th (Θ_10_), 30th (Θ_30_), and 50th (Θ_50_) percentile for the germinated fraction (*SE*: standard errors)

Species	Light–storage	*T*_b_	*SE*	*T*_c_	*SE*	*T*_o_	*SE*	Θ_10_	*SE*	Θ_30_	*SE*	Θ_50_	*SE*
*E. bivonae*	D–SS	7.8	0.22	26.1	0.17	17.0	0.12	34.3	1.53	36.3	1.67	41.7	2.11
	D–LS	7.6	0.52	32.4	0.51	20.0	0.28	29.1	2.29	30.9	2.51	33.8	2.88
	L/D–SS	8.4	0.16	26.4	0.15	17.4	0.09	42.1	1.55	48.6	1.95	54.1	2.36
	L/D–LS	9.0	0.96	31.7	1.08	20.3	0.62	23.7	4.56	25.9	5.25	28.5	6.17
*E. ceratocarpa*	D–SS	8.8	0.18	27.6	0.24	18.2	0.12	43.3	2.03	43.7	2.05	44.9	2.14
	D–LS	8.5	0.65	30.7	0.57	19.6	0.38	25.3	3.03	28.4	3.67	31.6	4.44
	L/D–SS	9.2	0.13	27.5	0.19	18.4	0.10	41.4	1.50	42.5	1.56	44.5	1.68
	L/D–LS	9.5	0.37	31.2	0.42	20.4	0.25	27.2	2.10	33.9	3.07	38.0	3.76
*E. characias*	D–SS	7.6	0.17	21.1	0.11	14.3	0.08	23.0	0.96	24.0	1.02	31.7	1.53
	D–LS	5.6	1.09	33.7	0.98	19.6	0.49	50.1	6.03	55.3	6.89	57.6	7.37
	L/D–SS	−0.5	3.98	34.3	1.79	16.9	1.47	91.2	19.49	91.9	19.66	99.9	22.0
	L/D–LS	6.8	0.41	32.7	0.37	19.7	0.21	47.1	2.56	49.3	2.75	53.7	3.13
*E. dendroides*	D–SS	9.1	0.47	30.6	0.45	19.9	0.29	22.6	2.07	28.0	2.97	33.1	4.08
	D–LS	7.9	0.51	34.7	0.77	21.3	0.35	26.8	2.10	29.6	2.50	32.3	2.88
	L/D–SS	8.7	0.15	28.7	0.25	18.7	0.12	23.2	0.80	26.9	1.01	31.3	1.29
	L/D–LS	7.9	0.34	33.4	0.42	20.6	0.20	27.0	1.44	29.5	1.65	31.8	1.91
*E. melapetala*	D–SS	8.9	0.18	23.6	0.39	16.3	0.18	18.8	1.23	21.3	1.53	28.3	2.24
	D–LS	11.1	1.15	30.2	0.51	20.6	0.56	18.9	3.10	19.1	3.19	19.5	3.31
	L/D–SS	4.2	0.86	25.2	0.27	14.7	0.41	61.5	5.19	61.5	5.19	65.1	5.71
	L/D–LS	10.6	0.75	30.1	0.30	20.4	0.36	19.8	1.86	21.0	2.03	22.6	2.30
*E. myrsinites*	D–SS	12.4	0.10	27.5	0.10	20.0	0.04	19.1	0.44	19.1	0.44	21.4	0.51
	D–LS	12.1	0.01	25.6	0.00	18.9	0.00	24.6	0.04	24.6	0.04	24.7	0.04
	L/D–SS	13.0	0.36	26.8	0.33	19.9	0.18	17.6	1.82	18.1	1.89	20.8	2.29
	L/D–LS	11.6	0.40	30.2	0.20	20.9	0.20	30.8	1.98	31.2	2.02	31.2	2.02
*E. rigida*	D–SS	6.2	0.57	27.1	0.40	16.6	0.04	25.5	2.05	28.4	2.36	29.8	2.56
	D–LS	7.0	0.51	31.1	0.37	19.1	0.00	32.2	2.28	34.8	2.60	38.7	3.08
	L/D–SS	6.7	0.81	27.2	0.64	16.9	0.18	24.1	3.10	25.7	3.40	27.7	3.80
	L/D–LS	7.6	0.69	32.6	0.71	20.1	0.20	30.2	3.19	32.3	3.55	35.4	4.07

Note

No germinations were observed at constant temperature for *Euphorbia amygdaloides* subsp. *arbuscula*.

Abbreviations: D, complete darkness; L/D, light/darkness (12/12 hr photoperiod); LS, long storage; SS, short storage.

Seed germination in response to temperature was well described by the thermal‐time model at suboptimal temperatures. Thermal times (°Cd) calculated for different germination percentiles (Θ_10_, Θ_30,_ and Θ_50_) are shown in Table 3. Values for Θ_50_ ranged between 20 and 100°Cd. The extremes were the *E. myrsinites*, which had a high *T*_b_ (ca. 13.0°C) and low thermal time (ca. 21 degree days), and *E. characias* which had a low estimated *T*_b_ (ca. 0.5°C) and a high thermal time (100 degree days).

## 4 DISCUSSION

Our results show that, in absence of other limiting factors (e.g., water), germination capability of the eight *Euphorbia* species was significantly influenced by temperature regimes, light conditions, and storage times, although the effects were species‐dependent.

In more detail, for all these species, except *E. amygdaloides* subsp. *arbuscula*, results show that the final germinated proportions were improved by a long storage period (>150 days). This finding supports the idea that most of these species are characterized by primary dormancy, which is released following an after ripening period (nondeep Physiological Dormancy; PD). Narbona, Arista, and Ortiz (2007b) have documented that also the seeds of *E. boetica* are affected by nondeep physiological dormancy. This might be regarded as an adaptation mechanism, by which germination is prevented immediately after seed dispersal, in May–June, when the season is becoming unfavorable for seedling establishment, and postponed to the beginning of the rainy season (García‐Fayos, García‐Ventoso, & Cerda, 2000; Quilichini & Debussche, 2000).

We tried to classify the abovementioned dormancy, according to Soltani, Baskin, and Baskin (2017). In this respect, it is possible to note that storage time did not only influence germination capability, and it also increased ceiling temperatures for *E. bivonae*, *E. ceratocarpa*, *E. characias*, *E. dendroides*, *E. melapetala*, and *E. rigida*. Accordingly, these species should be classified in the Type 1 nondeep PD category. On the contrary, storage time did not affect ceiling temperature in *E. myrsinites*, at least for germination in complete darkness. This behavior might support the idea that this species should be included in the Type 5 nondeep PD category (Soltani et al., 2017).

Once primary dormancy has been released, in the absence of other limiting factors (e.g., water), seed germination of the eight *Euphorbia* species seemed to be mainly driven by temperature. The highest germination capabilities and speeds were reached at optimal temperatures levels, ranging from 14.3 to 21.3°C. Base temperatures ranged from 5.6 to 12.1°C, while ceiling temperatures ranged from 25.6 to 34.7°C. In Mediterranean regions, under present climatic conditions, these results support a germination peak from early autumn to early winter, while mid‐winter germination is prevented by temperatures below the base level requirements. Early spring germination is also possible, although germinations may progressively become more difficult as the season progresses, because of water shortage (Fenner & Thompson, 2005). In summer, high temperatures (above the *T*_c_ level) may prevent germinations, until the unfavorable conditions are overcome. These results confirm that cardinal temperatures for germination represent a mechanism of adaptation, by which a given species can match its germination timing to favorable conditions for seedling recruitment (Huang, Liu, Bradford, Huxman, & Venable, 2016; Qiu, Bai, BiFu, & Wilmshurst, 2010; Thompson, 1970). Furthermore, the evident reduction in the germinative performance at both high (≥30°C) and low (5°C) constant temperatures may also dictate the altitudinal and latitudinal limits for the geographical distribution of these species.

During the unfavorable periods (winter and summer), the germination of these eight *Euphorbia* species may be obstacled either by thermo‐inhibition or by thermo‐dormancy. Our results show that seeds of *E. bivonae*, *E. characias*, and *E. dendroides* were thermo‐inhibited at high temperatures (Horowitz & Taylorson, 1983), while seeds of *E. amygdaloides* subsp. *arbuscula*, *E. ceratocarpa*, and *E. rigida* showed the existence of some mechanisms of thermo‐dormancy. Likewise, low temperatures caused thermo‐inhibition in three of the species under investigation (*E. characias*, *E. dendroides*, and *E. rigida*), while they caused thermo‐dormancy in the other species. Thermo‐inhibition has been documented in many perennial species or winter annual species in warm climates (Thanos et al., 1989; Thompson, 1973; Washitani & Masuda, 1990), including some *Euphorbia* species (Narbona et al., 2007a). Thermo‐dormancy has been also documented in annual desert plants (Gutterman, 1990; Gutterman, 2002; Thanos et al., 1989).

We also tested the effect of fluctuating temperatures, and we observed that only *E. amygdaloides* subsp. *arbuscula* and *E. myrsinitis* were favoured, in respect to a constant daily temperature regime. For all the other species, fluctuating temperatures induced an improvement of germination only for the highest levels of maximum daily temperatures (>25°C). This is probably due to the fact that, with the 8/25 and 8/30°C regimes, the average daily temperature was, respectively, 16.5 and 19°C, which is close to the optimal temperature level for all species.

The effect of light conditions was smaller than that of temperature. However, the results showed that, at fluctuating temperatures, seeds of *E. amygdaloides* subsp. *arbuscula*, *E. bivonae*, *E. ceratocarpa*, and *E. melapetala* were favoured by dark conditions. Likewise, *E. characias* and *E. dendroides* were slightly favoured, especially at the warmest temperatures (8/25 and 8/30°C). A good germination capacity in dark conditions was also observed in other *Euphorbia* species, such as *E. heterophylla*, *E. esula*, and *E. nicaeensis* (Best, Bowes, Thomas, & Maw, 1980; Brecke, 1995; Narbona, Ortiz, & Arista, 2006). It is possible that this phenomenon can be related to their large seeds or processes of seed dispersal. Indeed, many *Euphorbia* species are diplochorous and they are characterized by a primary explosive dispersal system and a secondary ant dispersal system, aided by the presence of the caruncle. Ants may bury the seeds inside their nest or may abandon them outside, on waste piles. In these conditions, the capacity to germinate in darkness can be advantageous. Some of the examined taxa have been confirmed to be myrmecochores (i.e., *E. characias*, *E. melapetala*), while some others may potentially be so.

Considering the abovementioned thermal requirements, the germination of these species may be vulnerable to climate change, which may narrow the favorable germination season and affect the dynamics of plant populations. However, the high habitat heterogeneity of the Mediterranean regions could ensure the future persistence of these plant species at local scale, allowing their migration to more favorable nearby ecological niches. More specifically, the expected reduction of precipitation in summer and winter seasons and substantial temperature increase should be less harmful for plant species of the thermo‐Mediterranean zone (i.e., *E. dendroides*, *E. bivonae*), through a slight altitudinal rise of communities and species migration.

In conclusion, most of the eight species of *Euphorbia* under investigation showed physiological dormancy, which was released following an after ripening period (nondeep Physiological Dormancy; PD). Germination was mainly affected by temperature, with optimal levels ranging from 14 to 21°C. The light regime showed lower effects with respect to temperature. These common traits seem to be rather typical for plant species growing in Mediterranean regions. In agreement with the results of Skordilis and Thanos (1995) relating to seeds of *Pinus brutia* and *P. halepensis*, our results support the idea that all the *Euphorbia* species under investigation exhibit a “Mediterranean germination syndrome,” which may be regarded as an adaptation strategy to typical elements of Mediterranean climates, that is, autumn seasons with mild temperatures associated to the restart of the rainy period. As the consequence, for all these species, germination should mainly occur during the autumn season.

Apart from the above similarities, the eight species also showed several differences, for example, in terms of threshold temperatures. Furthermore, suboptimal or supraoptimal temperature levels gave thermo‐inhibition in some species or thermo‐dormancy in other species. All these differences between species in terms of germination requirements may support their specific adaptation to local ecological factors. Furthermore, they demonstrate that *Euphorbia* species have neither similar germination traits, nor niche preferences, nor germination strategies, which suggests that seed germination is not likely to be a phylogenetically conserved trait.

Climate change, impacting on ambient temperatures, can strongly influence the dynamics and emergence patterns of Mediterranean *Euphorbia* species, restricting their favorable germination season.

These aspects are to be taken in account when defining the best measures for species conservation, either in situ or ex situ, particularly when plants must be propagated for vegetation restoration as well as for low‐maintenance Mediterranean gardens (i.e., *E. characias*, *E. dendroides*, *E. myrsinites* and *E. rigida*).

## CONFLICT OF INTEREST

The authors declare that they have no conflict of interest.

## AUTHOR CONTRIBUTIONS

AC, SC, AM, and AR conceived the ideas and designed methodology; AC, SC, and AR collected the data; AO and AM analyzed the data; AC and AO led the writing of the manuscript. All authors contributed critically to the drafts and gave final approval for publication.

## Data Availability

The main dataset is available at https://doi.org/10.5061/dryad.m1b1k35.
